# Strategies of Power, Status, and Control Among Carers in Nursing Homes; Influence on Clinical Learning Environment: A Qualitative Study

**DOI:** 10.1177/23779608251406341

**Published:** 2025-12-09

**Authors:** Vera Louise Sørø, Bjørg Aglen, Arne Orvik, Sylvia Søderstrøm, Gørill Haugan

**Affiliations:** 1 Department of Public Health and Nursing, Faculty of Medicine and Health Sciences, 8018 Norwegian University of Technology and Science, Trondheim, Norway; 2 Department of Public Health and Nursing, Faculty of Medicine and Health Sciences, 620066 Norwegian University of Technology and Science, Ålesund, Norway; 3 Department of Neuromedicine and Movement Science, 8018 Norwegian University of Technology and Science, Trondheim, Norway; 4 Faculty of Nursing and Health Science, 158927 Nord University, Levanger, Norway

**Keywords:** clinical learning environment, nursing homes, qualitative study, apprentices, nursing students, other-zero level

## Abstract

**Introduction:**

In nursing homes, registered nurses, associate nurses along with physiotherapists and occupational therapists represent different levels and length of education which may induce a struggle for power, status, and control affecting the clinical learning environment.

**Objective:**

This study scrutinizes how closure strategies of power, status, and control among health professions may affect the clinical learning environment in nursing homes.

**Design and Methods:**

In a qualitative, explorative design a strategic sample was used to explore the experience of power, status, and control in three Norwegian nursing homes aimed at improving the clinical learning environment for healthcare students and apprentices. An interprofessional preceptor team and interprofessional learning teams were established in each of the three nursing homes, facilitating collaboration, competency development, and confidence among the preceptors. Data were collected by focus group discussions and field observations.

**Results:**

The findings revealed a formal and informal hierarchy based on status and power among the health professions which influenced the clinical learning environment. Controlling routines became a strategy for the associate nurses to gain control over one's own work, and interprofessional collaboration was replaced by parallel practices. The apprentices followed their preceptors in daily routines and care, while the nursing students followed theirs, mainly conducting professional planning and development. The clinical learning environment was affected by perceived status differences between the professions, particularly evident when the learners participated in the interprofessional learning teams.

**Conclusion:**

The findings disclosed challenges concerning interprofessional collaboration, communication, and quality in clinical practice, possibly due to a struggle for power, status, and control among health professions. This seemed to be mirrored in the clinical learning environment representing a negative impact on students’ and apprentices’ opportunities to experience interprofessional collaboration. Interprofessional preceptor teams and learning teams represent possibilities to improve learning conditions and facilitate basic nursing learning.

## Introduction

Norwegian nursing homes (NHs) are long-term care institutions providing inpatient, 24 h nursing care based on the Act on Municipal Health and Care Services (Helsedirektoratet, 2022). NH residents are characterized by multiple and serious health problems. Consequently, NH care requires well-qualified health professionals. Currently, NHs face severe difficulties in recruiting registered nurses (RN) and other health professionals. Therefore, it is crucial that NH health professionals experience a meaningful work situation, a decent workload, a good working environment, as well as a good clinical learning environment (CLE) (Cooke et al., 2021; Husebo et al., 2018). A CLE providing good learning experiences is beneficial for both healthcare students and apprentices and thus recruitment of the required competencies (Thunborg, 2016). The clinical training in NHs is organized as guided clinical training (RETHOS, 2019). NHs offer clinical learning situations involving opportunities to learn from and about different health professions, shared knowledge, and role models. Through communities of practice in a local context of action, students and apprentices follow their discipline-specific preceptors (Thideman, 2005).

In Norwegian NHs, clinical staff consists of RNs, physiotherapists, occupational therapists, etc. holding a university bachelor's degree and associate nurses (ANs) representing a profession with a defined responsibility (Dahl, 2021; Flermoen, 2001; Heiret & Ludvigsen, 2012). The ANs hold a 2-year education in upper secondary schools followed by a 2-year apprenticeship supervised by ANs. The RNs are in charge and responsible for the resident care, and physiotherapists and occupational therapists work on assignments by the RNs. In Norwegian NHs, the nursing staff typically contains one-third RNs, one-third ANs, and one-third nurse assistants lacking formal healthcare education. Hence, the RNs represent a lesser part of the caring staff (Saunes et al., 2020; Ågotnes, 2017). RNs, ANs, and assistants take on different but complementary roles; good nursing care depends on collaboration between them.

## Literature Review

Interprofessional collaboration seems scarce in NHs; largely, the health professions perform their tasks alongside each other (Ardyansyah et al., 2024; Boon et al., 2004; Steihaug et al., 2016). Therefore, the best possible healthcare suffers (Corazzini et al., 2015; Ellström, 2010). Professional development which is about translating existing knowledge into practice, is hampered when interprofessional collaboration is scarce (Meld. St, 2022-2023). Competency development in NHs does not reflect the need of interprofessional collaboration (Grut et al., 2020; Office of the Auditor General of Norway, 2016; Bing-Jonsson et al., 2015).

Like the distinction between RNs and physicians concerning care and cure (Aase, 2016; The Joint Commission, 2008), a hierarchical distinction exists in NHs, dividing between the RNs who mainly develop and plan the care, versus ANs and nurse assistants who mainly perform the care (Grace, 2020; Sørø et al., 2021; Svendesen, 2019). This distinction involves a formal and informal hierarchy; formally by length and level of education followed by specific responsibilities for physicians, RNs, physiotherapists, and occupational therapists, and informally by the AN's extention of control over daily routines at the wards (Karlsson et al., 2008). Several routines in NHs are multifaceted regulating everyday life and routines often emerge unsystematically (Grace, 2020; Svendesen, 2019). Accordingly, routines rarely represent reflected actions but emerge from repeated ways of doing the work. Largely, routines represent a common perception of “how we do it here.” Consequently, clinical studies for students and apprentices are regulated by how daily routines are carried out, and by whom. During their clinical studies in NHs, these learners are socialized into the existing way of distributing the tasks between the professions at the ward (Svendesen, 2019; Karlsson et al., 2008).

Studies suggest that hierarchical and status differences lead to barriers to interprofessional collaboration, as well as recognition among the different professions in NHs (Braithwaite et al., 2013; Dahle, 2003). These barriers may negatively influence care quality, efficiency and working environment, and thereby recruitment and thriving in NHs (Farchi et al., 2023).

## Objective

This study scrutinizes how closure strategies of power, status, and control among health professions in NHs may affect students’ and apprentices’ CLE.

## Conseptual Framework

### NH Care—Organized by Routines

In NHs, care is organized by several *routines*, such as taking care of basic needs along with medical treatment. The routines are established to ensure resident safety and wellbeing, fulfillment of tasks and to work efficiently. A daily routine is characterized by: (1) a certain sequence of actions, thoughts, experiences and (2) the repetition of action patterns in a social context (Reckwitz, 2002). Generally, routines are established and strengthened over time (Reckwitz, 2002).

### Interprofessional Relationships—Closure Strategies

**Figure 1.**
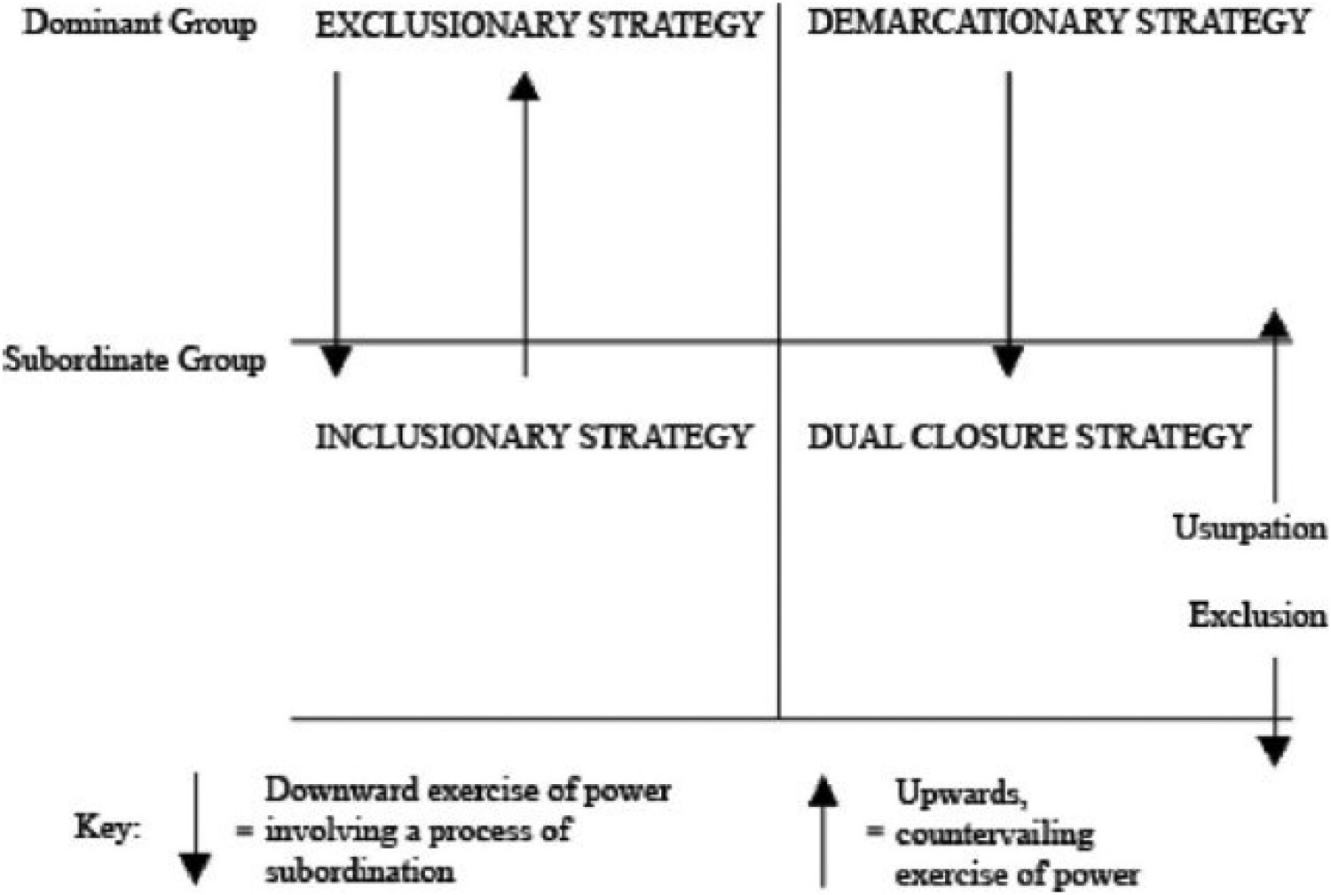
Strategies of closure: A conceptual model (Witz, 1992, p. 4). Reproduced with permission from The Licensor through PLSclear.

Looking at NHs, Witz's (1992) theoretical model portrayed in Figure 1 illustrates how struggling for status may induce power mechanisms and closure strategies among health professions. Witz depicts the interaction between professions on different levels based on their own traditions and strategies, urging them to defend and even raise their professional status. Figure 1 portrays Witz’ model (Witz, 1992) which conceptualizes both individual and collective closure strategies used in the interprofessional relationships in healthcare (Dahl, 2021). Four closure strategies characterize the interprofessional relationships: (1) exclusionary, (2) demarcationary, (3) inclusionary, and (4) dual. *Exclusionary strategies* may be downward use of power by RNs to control entry to their professional responsibilities and working tasks. *Demarcationary strategies* can be understood as RNs’ use of downward power to control the boundaries between related professions, in NHs mainly the ANs. RNs represent the dominant profession. Hence, the ANs’ strategies to entry into the rank of RNs can be seen as *inclusionary strategies*. Finally, *dual closure strategies* are directed both upwards and downwards; countervailing exercises of power intended to establish monopoly in an area, for example, the daily routines or care planning and development.

The left side of Figure 1 refers to interprofessional control by means of exclusionary as well as inclusionary strategies, while the right side illustrates professionals’ demarcation and dual closure strategies. Demarcationary strategies involve that the dominant profession (here, university educated) protects and consolidates its position of power. This can be seen as exclusion of ANs from care planning, while dual closure strategies are used to consolidate power, for instance by controlling who are allowed to take responsibility for what and to which time. Witz's (1992) theoretical perspective seems helpful in understanding the relationship between health professions in NHs (Dahl, 2021), and thus, to elucidate the presented data.

Summing up, care quality in NHs requires competency development and interprofessional collaboration. Even so, existing hierarchical distinctions seem to thwart the needed collaboration between the health professions. Knowledge is missing about how this hierarcical distinction, involving status, control, and power between health professions impacts care quality, the working environment, and thereby the CLE.

## Methods

### Design

In a qualitative, explorative design (Patton, 2015) a strategic sample was used to explore the participants’ experience of power, status, and control in NHs.

### Research Question

How does struggle for power, status, and control among health professions in NHs affect the CLE for students and apprentices.

### Sample and Settings

An interprofessional preceptor team (IPPT) was established at each of the NHs consisting of 16 professionals (7 RNs, 6 ANs, 2 physiotherapists, and 1 occupational therapist) supervising students and apprentices in the participating NHs; the IPPT teams met monthly for 2 h sessions (Aasen, 2014). The intention of the IPPTs was to facilitate interprofessional collaboration, competency development, and confidence among the preceptors and thereby improve the CLE for healthcare students (nursing, physiotherapy, and occupational therapy) and apprentices. In addition, an interprofessional learning team (IPLT) was established at each of the three NHs. The three IPLTs included students and apprentices and were led by the preceptors participating in the IPPTs. During the IPLT meetings, each IPLT evaluated and modified treatment plans for two patients at the specific NH.

Qualitative data were collected by means of five focus group discussions (FGDs) and field observations. The purpose was to study the preceptors’ experiences of participating in the IPPTs, and their observations of and reflections on students’ and apprentices’ interactions in the IPLTs. While Table 1 presents the wards and preceptors participating in the IPLTs, Table 2 lists the number of preceptors invited into and participating in the five FGDs. The field research included participating observations of the CLE at the three NHs.

**Table 1.** Number of Wards and Preceptors Participating in the IPLTs at NHs 1, 2, and 3.

	NH 1	NH 2	NH 3	Total
Wards	1	2	2	5
Preceptors of nursing students	2	4	1	7
Preceptors of apprentices	2	1	1	4
Preceptors of physiotherapy students	1	2	1	4
Preceptors of occupational therapy students	0	2	1	3

IPLT= Interprofessional Learning Team; NH=Nursing Home.

**Table 2.** Preceptors in the Five FGDs (All Were Woman).

FGD	Invited	Participated
FGD 1: Preceptors of students	9	6
FGD 2: Preceptors of apprentices	7	3
FGD 3: Preceptors of students and apprentices	7	5
FGD 4: Preceptors of students and apprentices	6	5
FGD 5: Preceptors of students and apprentices	6	4

*Note*: FGDs = Focus Group Discussions.

### Data Collection

The study aimed to examine how closure strategies of power, status, and control among health professions in NHs may affect the CLE. Data were collected during 2018 using the principles of FGDs. This method is relevant for investigating attitudes and experiences within a field, and to explore what, how, and why people think about a specific topic. The authors developed a semistructured guide using the principles of FGDs (Liamputtong, 2016) offering a provisional schedule of the time and the content of the FGDs; how it should be led and by whom, and potential themes to discuss. By giving the participants opportunities to express, form and modify their opinions during the discussion, group dynamics can facilitate the generation of original information and show how opinions are being built (Albanesi, 2024). Thus, the group dynamics in the FGD interaction process can clarify views that might be less accessible in individual interviews (Kitzinger, 1994; Krueger & Casey, 2009).

In this study, five FGDs (Table 2) were conducted involving 23 preceptors. Participants in two of the FGDs were preceptors for either nursing students or apprentices, while the three other FGDs were interprofessional including preceptors for both students and apprentices. In general, asymmetrical relations in FGDs should be avoided. Participants should be homogenous enough to feel “among peers” and comfortable enough to express their opinions (Albanesi, 2024). However, homogenous groups entail a risk of groupthink (Roller & Lavrakas, 2015). In heterogeneous groups, particularly when guided by moderators, different opinions can emerge and stimulate discussions and deeper insight.

In their roles as preceptors in the clinical learning environment, the FGD participants can be involved in issues of power imbalances and hierarchical structures. Particularly, such issues can appear in the three FGDs with an interprofessional approach. However, all participants were experienced as professionals. Group members’ knowledge and practice within the field being discussed in FGDs is a significant contextual factor in organizing groups and analyzing findings. Though, the moderators should be particularly aware of and handle potential tensions. The second author led the FGDs while the first author took notes and made the transcriptions. On average, the FGDs lasted 1.5 h, and sound recordings were made. The discussions were facilitated by inputs such as “please, talk about your experiences being a preceptor here,” and the following discussions allowed new themes to emerge.

The second author conducted 2 days field work with 8 h observation pr day in the three NHs: the field notes include participating observations of the CLE and care organization, learning situations, preceptorship, and learning rewarded by managers, along with notes from conversations with the managers. A guide for participatory observation (Lofland & Lofland, 2006) was used to guide and structure the researcher's role in the field. Field notes were conducted immediately after each observation session (Creswell & Poth, 2018).

### Inclusion Criteria

The participants in this study were experienced both as professionals and as preceptors for either nursing students or apprentices.

### Ethical Considerations

A convenient sample of three public NHs agreed to participate: one rural and two urban. Employees (Table 1) were then surveyed. The participants, all experienced with interprofessional teams, received oral and written information about the purpose of the study as well as information about confidentiality and their right to withdraw at any time. The study was conducted in accordance with the Declaration of Helsinki and approved by The Norwegian Agency for Shared Services in Education and Research (NSD).

### Analysis

Field notes and FGD transcripts were constantly consulted for comparison throughout the analyzing process. Using Braun and Clark's (2022) six-step process of reflective thematic text analysis (RTA), identification, analysis, and interpretive patterns of themes within the data were emphasized. The rationale for choosing RTA was to enable critical reflections on our roles as researchers, research practices, and processes. In phase 1, the data were reviewed several times for latent meaning and patterns across the datasets and notes were made simultaneously. Phase 2 included initial coding, systematic review of the data, and coding of meaningful units. Then, relevant data were sorted, setting aside irrelevant data, noting thoughts, ideas, and reflections. Phase 3 involved critical interrogating and unpacking meaning around themes and issues. The codes were examined, and the data were compared to identify a broader meaning and potential themes. Thereafter, the codes were analyzed to find broader overarching themes. The codes that did not fit into any theme were temporarily discarded. In phase 4, the most relevant themes were selected. Data within each theme were then evaluated regarding meaning in relation to each other and whether they differed from other themes. Initial coding were reviewed in case something was overlooked. In phase 5, the themes were defined, named, reviewed, and examined for any weaknesses before they were named based on the theme content. In the sixth and last phases, the analytical narratives and data were put together to provide a comprehensive description and contextualization in relation to relevant literature. All authors were involved in all phases: the first author drafted a preliminary text which the authors reviewed and discussed. Table 3 exemplifies four of the six phases. Phase 5 involved making a summary of each topic and determining the topic names, while phase 6 was reporting. The analysis was conducted with an inductive approach involving gathering data searching for patterns (Bingham, 2023).

**Table 3.** Exemplification of the Analysis.

**Step 1** Naive reading	*Search for meaning and patterns*
**Step 2** Initial coding of meaningful units	*Preceptors for nursing students:* “ANs have less value.” “Students are university, apprentices are upper secondary school; students are students, apprentices are also labor.” *Preceptors for apprentices:* “ANs have less value when priorities are set.” “The apprentices experience that they have nothing to bring; the students easily take the leadership role.” “Students are students, apprentices are apprentices.”
**Step 3** Condensation—searching for themes	ANs and their apprentices experienced being the lowest ranked in the hierarchy of trained health professionals. This experience was also supported by other trained health-professionals
**Step 4** Selection of themes relevant to the aim	A formal and informal hierarchy of status and power at the wards affected students’ and apprentices’ CLE

ANs=Associate Nurses; CLE= Clinical Learning Environment.

## Reflexivity and Transparency

Researchers’ competencies and experiences are likely to influence the interpretation of data (Braun & Clarke, 2022). Qualitative research involves researchers becoming instruments for collecting, interpreting, and analyzing data. Thus, to move beyond preconceptions and develop new and deeper understandings, the researchers must identify, define, and reflect on their own preconceptions of the topic. Providing a brief description of the researchers’ preunderstandings is crucial for transparency (Marshall & Rossman, 2016, pp. 43–62). In this study, the researchers were of both sexes, four registered nurses and one physiotherapist, all having experience in clinical NH care as well as being university professors experienced in teaching students about NH care. The researchers’ preconceptions in this study involved the fact that NH care is mainly based on a hierarchic structure, and tension between the different caring professions. In accordance with the framework of Braun and Clarke (2022), the transcripts were first read by one researcher to establish a preliminary familiarity with the data. Thereafter, each transcript was examined multiple times by two researchers to identify emerging patterns. During this initial phase, the data suggested that issues of care provision were a source of tension between RNs and ANs, with indications of struggle for power and a quest for professional recognition among these groups. Subsequently, the transcriptions were analyzed holistically to enable a more comprehensive interpretation of the dataset. To enhance methodological rigor and ensure trustworthiness, the research process was operationalized through a structured, multistep design of data collection, analysis, and reporting, consistent with established qualitative standards (Johnson et al., 2020; Jootun et al., 2009). The steps implied that (1) all researchers were involved in study design as well as creating research questions, (2) all authors carried out a joint analysis and interpretation of all data, after which (3) the themes and study structure were chosen. Step 4 consisted of joint reviews to ensure agreement on interpretation of the interviews included, whereas (5) possible biases were assessed, and finally (6) the NHs as study context were highlighted throughout.

## Results

This study scrutinizes how possible closure strategies of power, status, and control among the health professions in NHs may affect students’ and apprentices CLE.

### A Formal Hierarchy and an Informal Hierarchy of Status and Power at the Wards

In the NH context, professionals with a university degree achieved the highest status at the wards. Upper secondary educated professionals representing a lower level as well as shorter education did achieve power, but without the same status; they controlled when and how the daily routines were carried out.

The data showed that each professional group seemed to protect its domain by controlling one's work without interference by other professional groups. Hence, the data uncovered an ongoing struggle for power and control among the professions. For example, this struggle concerned controlling the various tasks and routines. By doing so, the ANs maintained pressure against those with university education, while the university group defended their status. Preceptors for students along with preceptors for apprentices disclosed that this struggle for status and power affected the CLE and thus the learners; “*Students are university graduates, with another status. Apprentices come from upper secondary school”* (preceptor of students, FGD 2). “*Students are students, apprentices are apprentices*” (preceptor of apprentices, FGD 1).

Accordingly, a similar struggle for status and power could be seen among the learners. Those precepting the apprentices participated in the IPLTs, however acknowledging that “*the apprentices experience that they have nothing to bring, the students easily take the leadership*” (FGD 5). Another preceptor ensured that “*apprentices look up to the nursing and physiotherapy students*” (FGDs 1 and 5), and “*the students listen to those with a university education, not to the associate nurses*” (FGD 5), indicating that the ANs did not achieve the same acknowledgement and power which is also reflected in the CLE. The participants perceived a difference in status between ANs at the one side, and RNs, physiotherapists, and occupational therapists at the other. Consequently, this issue of status affected the CLE and thus the learning possibilities. A preceptor for students stated that “*students have their own status*” (FGD 2), while a preceptor for apprentices said, “*apprentices are set to work*” (FGD 1). This corresponded to the general experience of several preceptors of apprentices; “*it should be important what also apprentices have to contribute*” (FGDs 4 and 3) and “*there is a big difference in who the students listen to; the students don’t listen to the apprentices*” (FGD 5). This was stated by students and emphasized in IPLTs and overheard by several preceptors of apprentices.

The ANs controlled and carried out most of the daily routines, and all other work had to be adapted. For example, “*doctor's visit cannot take place before 08.00 am*” (field observations), “*the occupational therapist cannot arrive until the patient has had breakfast*” (field observations). What's more, the distribution of tasks played out in the CLE through control over the routines. This also involved which profession was responsible for what. These conflicts about routines appeared to be associated with workload demands, variability in tasks, and the ANs’ feelings of lack of respect.

The ANs experienced that their profession was expected to constantly conduct the “heavy” work, along with implementing what the RNs decided.

### Distribution of Tasks as a Strategy to Maintain Control Over Own and Others’ Work

The ANs wanted an even distribution of basic nursing work. However, at the same time they kept protecting “the heavy work” as “their” area. The ANs also maintained control over basic nursing routines by resisting changes entailing additional tasks to be conducted, for instance implemented by the physiotherapists or RNs. The ANs seemed sensitive to signals indicating a devaluation of their role and value. For example, they disliked not being invited to interprofessional meetings, because “*we are the ones who really know the residents*” (preceptors for apprentices, FGD 5). Despite a general agreement that the ANs really knew the residents, during interprofessional meetings the ANs were not included since they should rather care for the residents. Accordingly, the ANs felt devaluated. The perceived difference in status and value was maintained because the professions kept to “their” working tasks and specific responsibilities. A physiotherapist said, “I *feel that RNs stick to their own profession*” and continued, “*ANs are not allowed to participate in interprofessional meetings*” (FGD 5). The other professions supported the ANs’ intention to attend interprofessional meetings concerning basic nursing care. Though, for reasons mentioned above, the ANs were not included in such meetings.

When RNs stuck to their own profession, ANs carried out the daily care and decided how the different tasks should be carried out and how much time each task should take. Largely, students and apprentices followed their main preceptors and their work at the wards. Consequently, the distribution of roles and the perceived differences between the professions were maintained also in the CLE.

### Interprofessional Collaboration is Replaced by Parallel Practices

The data revealed that basic nursing care was not a topic of discussion among health professions. Instead, they focused on “*how bad they feel in their everyday working situation*” (FGDs 1–5) and “*how difficult it is to get the work done*” (FGDs 1–5). They did not discuss clinical nursing and consequently excluded it from becoming a topic in the CLE. Largely, it seemed that everyone was concerned with the perceived difference of status between the professions at the wards.

Correspondingly, interprofessional collaboration was scarce. Different approaches to holistic care were utilized without any collaboration or discussion. Thus, this did not become an important topic in the CLE. Although the different professions stated that more interprofessional collaboration was warranted, it didn’t happen: “*RNs stuck to their own profession*” (physiotherapist) (FGD 5). An RN commented that “*apprentices should also take part in the interprofessional meetings*” (FGDs 4 and 5), supported by an AN stating that “*apprentices should take part in the interprofessional meetings*” (FGDs 4 and 5). Another AN stated “*I have hardly had any meetings with those who precept students. We should have such meetings*” (FGD 1). This was supported by another AN; “*tips and advice from other professions’ perceptions are important*” (FGD 1).

All these statements underline a general experience that interprofessional collaboration was limited with implications for the CLE.

## Discussion

This study aimed to scrutinize how closure strategies of power, status, and control among health professions in NHs may affect healthcare students’ and apprentices’ CLE. According to Witz (1992), among other things closure strategies describe the responses of subordinated groups using upwards, countervailing exercises of power when affected by demarcationary strategies by the dominant groups. This strategy is important to understand the perspectives of professions and the power mechanisms among them, all of which are significant to care quality and the CLE.

### A Formal Hierarchy and an Informal Hierarchy of Status and Power

The findings revealed the existence of a formal hierarchy and an informal hierarchy based on perceived status among the professions, ranking the university educated on top. Concurrently, the ANs searched for power by controlling the distribution of work which was also a strategy to cope with a stressful workday. This hierarchy was echoed in the learners’ attitudes toward each other and the preceptors, entering the same status level as their preceptors. University education conferred higher status. This distinction was mirrored in the IPLTs, where apprentices claimed students were more worth than themselves. Accordingly, the students took the lead, while the apprentices adhered to that; “*the apprentices listen to those with a university education, not to the ANs*” (IPLT-preceptor of apprentices).

Mostly, ANs’ responsibilities involved handling the daily routines, while RNs, physiotherapists, and occupational therapists developed and planned the care. Possibly, the ANs perceived that RNs decided over them, and thereby impacted on their working day and making their work less predictable. Or the opposite, very predictable; to the boring point, doing the same things every day. The RNs also imposed extra work on the ANs by their planning and quality development. In response, the ANs opposed by taking ownership of the routines and they accepted no interferences. Considering the limited resources in NHs, the ANs are concerned with surviving with a heavy workload; “*Time is too short*” (FG 1, preceptor of apprentices) and “*time is a challenge*” (FG 2, preceptor of students).

Nevertheless, this attitude was mirrored among students and apprentices; “*apprentices are used as manpower*” (FGs 1 and 2). The ANs protected their caring and routine domains to control their workload. Dominating the routines was a strategy echoed in previous research showing that professions are fighting for influence on decisions concerning patient care, and thus the working tasks (Baker et al., 2011). The ANs’ strategies to achieve and preserve their status can be seen as an attempt to highlight their position. By mastering basic care, including how the routines of care should be carried out, they seek recognition and appreciation (Dahle, 2003; Liu, 2015).

### Distribution of Tasks as a Strategy to Control One's Work Situation

In this study, the ANs used inclusionary strategies (Witz, 1992) toward the RNs to maintain control over their work by dominating the routines. The same goes for the RNs striving for care quality, etc. Hence, parallel practices emerged. As a result, working tasks and routines became important to the apprentices, while reflections, discussions, and quality development seemed important only for students. In its ultimate consequence, students’ and apprentices’ motivation for learning and wellbeing may be reduced, inhibiting recruitment to the NH sector: routine work and power struggle win over professional development and community. Professional development is the means of enhancing competencies in NHs (Yu et al., 2022). However, the struggle for power controlling different aspects of the CLE such as routines and quality development seemed to maintain status quo. Due to the distinction between the RNs and ANs, performance of routines can be rigid, that is, carried out by ANs in their way (Bach et al., 2012; Kessler et al., 2019); consequently, evidence-based care may be lacking.

### RNs Stick to Their Own Profession and Tasks

The distinction of working tasks/responsibilities between the professions is consistent with studies showing that RNs, physiotherapists, and occupational therapists stick to their duties, while ANs stick to their domains (Fryer et al., 2016; Jansen et al., 2017; Shanagher, 2017). According to Witz (1992), this distinction can imply demarcation and other closure strategies among the professions: implementation of changes is difficult in working cultures where struggle for power and status maintains, accompanied by a consolidation of the existing hierarchy (Dahle, 2003; Langley et al., 2019; Witz, 1992).

Hence, the ANs risk being most concerned with maintaining existing status differences between professions and “surviving in daily work” rather than participating in discussions on how to secure care quality. On the other hand, also RNs, physiotherapists, and occupational therapists support the existing status differences between the professions when they seize the development aspect and do not actively participate in the daily resident care. Consequently, both professional and quality improvement suffer. According to Andreasen et al. (2020), an important issue for learners to remain in the healthcare system is the educational atmosphere in the CLE. Thus, the struggle for power and status among the professions may imply that the healthcare system loses important future workforce as the learners mostly experience consolidation of existing status differences rather than discussions on how to improve care quality.

### ANs are Not Included in Interprofessional Meetings

Research shows that interprofessional meetings are reserved for health professionals with a university degree, such as RNs, physiotherapists, etc. (Ingelsrud & Falkum, 2017; Drennan et al., 2018). Due to staffing, the ANs are mainly engaged in resident care during such meetings. Consequently, ANs experience less opportunities for collaboration and influence on resident care. What's left for them is the routines. This also affects the CLE as apprentices are excluded from the interprofessional meetings while students participate. Evidently, this way of organizing care implied a loss of information and care quality as ANs’ knowledge and insights about the residents are vital (Coghill, 2018; Lindh et al., 2018). Instead, the distinction between university graduates and upper secondary educated is maintained and perhaps reinforced. Looking at the organization of the CLE, the status differences are likely to be maintained: apprentices follow ANs while students follow RNs, etc., adapting to the status differences. In addition, professional development may be at a standstill, as practice does not follow theory and evidence: some do the planning, while others implement and conduct.

Our data showed that the ANs feel undervalued, downgraded, and ignored, which is in line with findings in other studies (Cronin et al., 2020; Phillips et al., 2008; Young et al., 2017). Excluded from interprofessional meetings about planning and quality development, the ANs in this study felt dissatisfied, with a reduced self-confidence. Such experiences do not support interprofessional collaboration and may hamper care quality and the CLE. Furthermore, ANs may feel abandoned when RNs do not participate in the daily care but essentially leave it to them. This may indicate a certain lack of confidence in one's own competence (Drennan et al., 2018). The findings disclosed a dearth of self-confidence among apprentices in the IPLTs (FGD 5, p. 11), indicating that distrust in one's competency possibly spilled over from the ANs to their apprentices.

### Interprofessional Collaboration is Replaced by Parallel Practices

Interprofessional collaboration in the three NHs seemed scarce; the professions rather worked in parallel practices. Consistent with previous studies (Franklin et al., 2015; Tsakitzidis et al., 2017), each profession seemingly worked on their own tasks. Consequently, available knowledge may not be shared which is pointless, since interprofessional collaboration is necessary to increase professional competencies (Beynon et al., 2022; Wei et al., 2022). In this study, the learners were socialized into a caring culture with minor interprofessional collaboration. As a result, this way of working remains with absence of interprofessional collaboration. The CLE might be experienced as less fruitful as well as not very interesting.

## Strengths and Limitations

All authors regularly discussed the data, interpretations and findings. However, the present data typically represent Norwegian NHs, which may diminish the transferability to NHs worldwide. Beforehand, there was a well-functioning collaboration between the university and these NHs, representing a strength of this study. Furthermore, the researchers were nurses and physiotherapists with extended experience of NHs as a clinical field and learning environment for healthcare students. The same goes for the researcher who conducted the observations and took the field notes. The use of an observation guide helped to structure the observations and ensured breadth and depth of the data. The transcription of observational data was conducted by the researcher herself. Moreover, the two researchers who conducted the FGDs made the transcriptions immediately after the interviews, ensuring that important interpretations and understandings of the recorded data were noted. Considering the strong preunderstandings of the researchers, the members of this research group held different positions: those who collected the data and made the transcripts represent a close relation to the data and the underlying meaning, while the other researchers held a more distant position, ensuring an open approach to the interpretation of the data.

## Implications for Practice

When implementing organizational changes in NHs, it is imperative to foster interprofessional dialogue grounded in collaborative engagement, trust, and mutual respect for professional competencies. Consequently, structural adjustments to existing work practices appear necessary to support sustaining models of care. Specific implications for nursing primarily concern the development of strategies that strengthen professional identity while contemporarily promoting collaborative decision making and shared responsibility in an interprofessional context.

## Conclusions

The strategy among the ANs was to control the routines: however, controlling routines became a strategy for power and control more than a strategy for the best resident care. This strategy was mirrored in the CLE, and the CLE suffered. If routines are utilized for surviving in daily work, this can be critical for the working environment and care quality. Consequently, all stakeholders are at the risk of decline, including the learners. These mechanisms impacted negatively on students’ and apprentices’ opportunities to learn from and about different health professions. Looking at interprofessional collaboration in the light of power, control, and status, it seemed that power resulted from status and controlling the work tasks, leading to manifestation of hierarchy among the health professions. In NHs, university graduates possess a higher status and are responsible for planning and development activities. Students and apprentices encounter, learn, and adapt to this hierarchical distinction. Hence, these learners are trained and socialized into a CLE based in hierarchical distinction with parallel practices.
